# Jasmonic Acid and Salicylic Acid improved resistance against *Spodoptera frugiperda* Infestation in maize by modulating growth and regulating redox homeostasis

**DOI:** 10.1038/s41598-024-67151-1

**Published:** 2024-07-22

**Authors:** Bilqees Kanwal, Samina Tanwir, Farooq Ahmad, Jam Nazeer Ahmad

**Affiliations:** 1 Plant Stress Physiology and Molecular Biology Lab, Department of Botany, University of Agriculture Faisalabad, Faisalabad, Pakistan; 2 Department of Entomology, University of Agriculture Faisalabad, Faisalabad, Pakistan

**Keywords:** Phytohormones, Herbivory, Defensive response, Hydrogen peroxide, Proline, Phenolics, Physiology, Plant sciences

## Abstract

Exploring host plant resistance and elevating plant defense mechanisms through the application of exogenous elicitors stands as a promising strategy for integrated pest management. The fall armyworm, a pernicious menace to grain crops in tropical and subtropical regions, stands as a formidable threat due to its capacity for devastation and a wide-ranging spectrum of host plants. There is no literature regarding artificially induced resistance in maize against fall armyworm (*Spodoptera frugiperda*) by exogenous application of phytohormones. The present investigation was performed to evaluate the role of jasmonic acid (JA) and salicylic acid (SA) on two maize hybrids namely FH-1046 and YH-1898 against fall armyworm. Results showed that plant height, biomass and lengths, fresh and dry weight of root shoot which decreased with armyworm infestation improved with phytohormonal application. JA treatment resulted in a higher increase in all attributes as compared to SA treatment. Improvement in relative water contents, photosynthetic pigments and pronounced levels of phenol and proline accumulation were observed in infested plants after JA treatment. Infested plants recovered from oxidative stress as JA application activated and increased the antioxidant enzyme activity of superoxide dismutase, peroxidase and polyphenol oxidase activity in both FH-1046 and YH-1898 . The oxidative stress reduction in infested plants after JA treatment was also evident from a fair decrease in MDA and H_2_O_2_ in both varieties. The SA and JA mediated genes expression was studied and it was found that in FH1046 maize cultivar, JA dependent genes, particularly marker genes PR1 and Lox5 were highly expressed along with TPS10 and BBT12. Whereas SPI, WRKY28, ICS and PAL were shown to be activated upon SA application. Evidently, both JA and SA elicited a robust defensive response within the maize plants against the voracious *S. frugiperda*, which in consequence exerted a discernible influence over the pest's developmental trajectory and physiological dynamics. A decrease in detoxification enzyme activity of the insects was observed after feeding on treated plants. Moreover, it was recorded that the survival and weight gain of FAW feeding on phytohormone treated maize plants also decelerated. In conclusion, FH-1046 was found to be more tolerant than YH-1898 against fall armyworm infestation and 1 mM JA was more effective than 1 mM SA for alleviation of fall armyworm stress. Therefore, it was inferred that phytohormones regulated redox homeostasis to circumvent oxidative damage and mediate essential metabolic events in maize under stress. To our current understanding, this study is the very first presentation of induced resistance in maize against *S. frugiperda* with the phytohormonal application (JA and SA).

## Introduction

Agricultural productivity is constantly challenged by a myriad of biotic stresses, with insect pests emerging as significant threats to global food security. There are 140 different insect pest species that attack maize (*Zea mays*) out of which 10 species are the most destructive ones^1^. Among these pests, the Lepidopteran fall armyworm (*Spodoptera frugiperda*) stands out as a formidable adversary relentlessly devastating maize crops across the world^2^. The economic and ecological consequences of such infestations are profound, urging a deeper exploration of strategies to mitigate the damage inflicted by these voracious insects. Originating from the tropical and sub-tropical regions of the United States of America, FAW moved on to Europe, Africa, Asia, and Australia^2^ becoming a worldwide threat to 353 crops of different families^3^. Although it likes to feed on a wide range of host plants like sugarcane, maize, wheat, millets, rice, tomato, cotton, groundnut, cabbage, green amaranth and pasture grasses. However, the pest has primarily caused significant economic losses to maize along with a few other crops which are preferred as a second option^4,5^. It was first reported in the Sindh province of Pakistan in various spring corn fields in March 2019. Fall armyworm can cause yield reduction due to its higher reproduction rate, polyphagous nature and ability to move long distances at night^6,7^.

**Figure.1:**
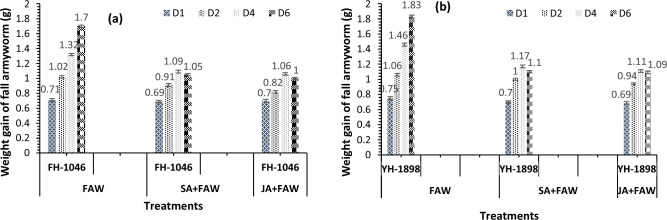
(1**a**, 1**b**) Fall armyworm weight gain (g) of two maize hybrids after infestation on control, jasmonic acid and salicylic acid application. Bars (mean ± SE) values are given.

**Figure.2:**
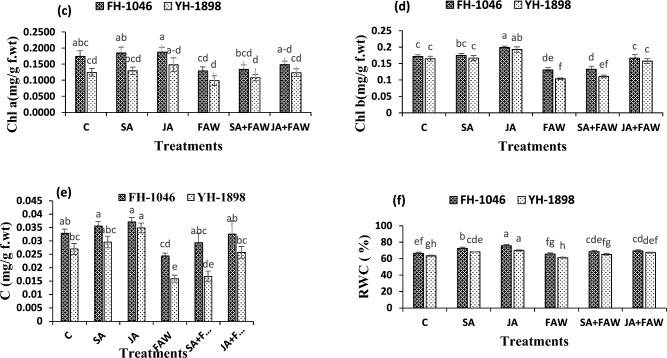
(2**c**, 2**d**, 2**e**, 2**f**.) Chlorophyll A, Chlorophyll B (mg/g f. wt), Carotenoids content (mg/g f. wt) and Relative water content (%) of two maize hybrids after fall armyworm infestation and jasmonic acid and salicylic acid application. Bars (mean ± SE) with similar letters are not statistically different.

**Figure.3:**
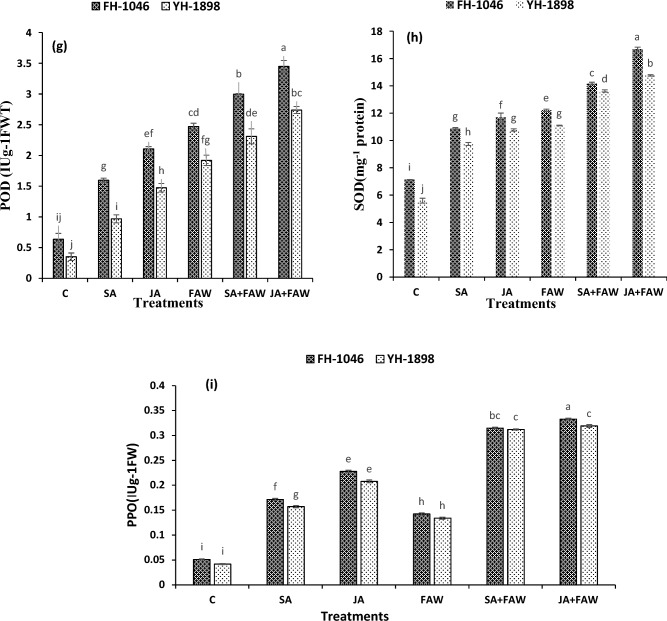
(3 **g**, 3 **h**, 3**i**) POD activity (IUg-1 FWT), SOD activity (mg-1 protein) and PPO content (µmol/g fwt) of two maize hybrids after fall armyworm infestation and jasmonic acid and salicylic acid application. Bars (mean ± SE) of same color with similar letters are not statistically different.

Maize, commonly known as corn, stands as a fundamental pillar of global food security. This cereal crop's adaptability and versatility has rendered it a staple food for billions, spanning diverse cultures and economies^8^. It fulfills about 30% of the food demand of in various forms providing several vitamins, fiber, starch, phytochemicals, oil and proteins that are crucial parts of our diet^9^. Additionally, it also contributes as raw material for diverse industrial products^10^. With the passage of time the demand for maize grains has increased due to the alarming rise in human population (Tables 1 and 2). However, maize's contribution to food security is threatened by the pervasive impact of insect pests, which can inflict substantial damage on crops, triggering economic losses and compromising the availability of this crucial dietary staple posing threats to sustainable food security. A huge amount of money is spent on insecticides employed to manage the *S. frugiperda*. (Fig. 1) Nonetheless, the excessive application of these agrochemicals has resulted in numerous ecological and environmental concerns (Fig. 2). These encompass issues like the development of insecticide resistance, resurgence of pests, emergence of secondary pests, accumulation of insecticide residues, and depletion of biodiversity within ecosystems. Consequently, the formulation of more secure pest control approaches, such as enhancing and harnessing the innate resistance of plants, becomes a crucial undertaking^11,12^ (Fig. 3).

**Table 1:** Plant height, shoot length, root length, shoot fresh weight, root fresh weight, shoot dry weight, root dry weight, flag leaf area of two maize hybrids after fall armyworm (FAW) infestation and foliar application of salicylic acid SA and jasmonic acid (JA).

Treatments	Genotypes	Plant height (cm)	Shoot length (cm)	Root length (cm)	Shoot fresh weight (g)	Root fresh weight (g)	Shoot dry weight (g)	Root dry weight (g)	Flag leaf area (cm^2^)
C	FH-1046	65 ± 0.47 bc	48.67 ± 0.72 d	31 ± 0.47 de	47.46 ± 0.49 e	17.67 ± 0.33 cd	4.29 ± 0.32 cd	1.99 ± 0.037 ef	206.98 ± 3.24 c
	YH − 1898	53.67 ± 0.72 fg	44 ± 0.47 f.	26 ± 0.47 g	41.09 ± 0.48 h	15.04 ± 0.54 gh	3.21 ± 0.33 fg	1.93 ± 0.036 f.	203.50 ± 3.10 c
SA	FH − 1046	67 ± 0.47 ab	54.67 ± 0.54 a	34.67 ± 0.54 ab	51 ± 0.72 b	20 ± 0.58 b	5.55 ± 0.33 b	2.29 ± 0.050 a	245.6 ± 3.08 a
	YH − 1898	57.33 ± 0.54 e	49 ± 0.47 cd	29 ± 0.47 e	47.5 ± 0.21 de	17.34 ± 0.88 de	4.01 ± 0.29 c-f	2.05 ± 0.034 def	231.05 ± 3.29 b
JA	FH − 1046	69 ± 0.47 a	55.33 ± 0.72 a	36 ± 0.47 a	54 ± 0.47a	21.73 ± 0.37 a	6.74 ± 0.34 a	2.33 ± 0.031 a	251.73 ± 3.12 a
	YH − 1898	59.67 ± 0.72 d	51 ± 0.47 bc	31.33 ± 0.54 cd	49.40 ± 0.32 bc	19.17 ± 0.56 bc	4.22 ± 0.33 cde	2.08 ± 0.030 cde	242.43 ± 3.54 a
FAW	FH − 1046	60.33 ± 0.72 d	50 ± 0.47 cd	29 ± 0.47 de	44.27 ± 0.29 g	16 ± 0.58 efg	3.30 ± 0.14 efg	2.13 ± 0.031 bcd	200.07 ± 3.21 c
	YH − 1898	52.33 ± 0.54 g	43 ± 0.47 f.	22.67 ± 0.72 g	39.13 ± 0.43 i	14 ± 0.57 h	3.06 ± 0.29 g	1.92 ± 0.035 f.	175.96 ± 3.00 d
SA + FAW	FH − 1046	64 ± 0.47 c	51 ± 0.47 bc	30 ± 0.47 de	48 ± 0.47 cde	18 ± 0.58 cd	4.64 ± 0.36 cd	2.20 ± 0.024 abc	225.03 ± 3.01 b
	YH − 1898	55.33 ± 0.72 ef	46 ± 0.47 e	26 ± 0.47 f.	45.25 ± 0.56 fg	15.67 ± 0.33 fg	3.22 ± 0.61 fg	2.06 ± 0.042 def	205.25 ± 2.95 c
JA + FAW	FH-1046	66.33 ± 0.72 b	52.33 ± 0.54 b	33 ± 0.47 bc	49.33 ± 0.54 cd	19.73 ± 0.37 b	4.76 ± 0.35 bc	2.25 ± 0.031 ab	228.30 ± 3.20 b
	YH − 1898	57 ± 0.47 e	48.67 ± 0.54 d	28.33 ± 0.27 e	46.65 ± 0.27 ef	16.41 ± 0.30 def	3.82 ± 0.33 d-g	2.08 ± 0.052 cde	207.58 ± 3.47 c
Anova	G	**	***	***	***	**	***	***	**
	TRT	***	***	***	***	***	***	***	***
	G*TRT	NS	NS	NS	***	NS	**	NS	*

Significant differences at p ≤ 0.05, 0.01, 0.001 are denoted with the help of *, **, *** respectively. NS is used to show non-significant differences at p < 0.05.

Means ± standard error values in columns denoted by different letters vary significantly from one another at p ≤ 0.05.

**Table 2:** carboxyl transferase and glutathione S transferase activity in FAW and SA + FAW and JA + FAW.

TRT	G	Carboxyl transferase	Glutathione S transferase
FAW	FH-1046	344 ± 2.1 b	1192 ± 6.9 c
	YH-1898	378.7 ± 3.0 a	1358 ± 7.4 a
SA+FAW	FH-1046	250.7 ± 2.3 d	1157.7 ± 7.2 d
	YH-1898	303.3 ± 2.4 c	1287.7 ± 7.3 b
JA+FAW	FH-1046	176.3 ± 2.6 f	982.7 ±7.2 e
	YH-1898	188.7 ± 2.6 e	995.3 ± 8.1 e
**Anova**			
	G	***	***
	TRT	***	***
	G*TRT	***	***

Significant differences at p ≤ 0.05, 0.01, 0.001 are denoted with the help of *, **, *** respectively. NS is used to show non-significant differences at p < 0.05.

Means ± standard error values in columns denoted by different letters vary significantly from one another at p ≤ 0.05.

**Figure.4:**
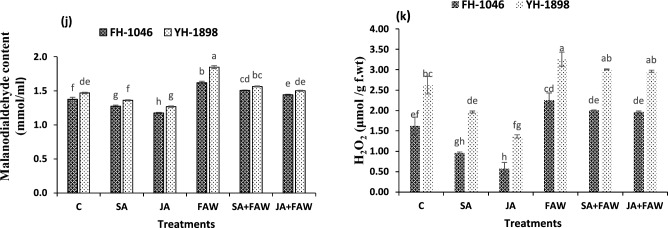
(**j**, **k**) MDA (m mol /ml) and H_2_O_2_(µ mol /g fwt) of two maize hybrids after fall armyworm infestation and jasmonic acid and salicylic acid application. Bars (mean ± SE) of same color with similar letters are not statistically different.

**Figure.5:**
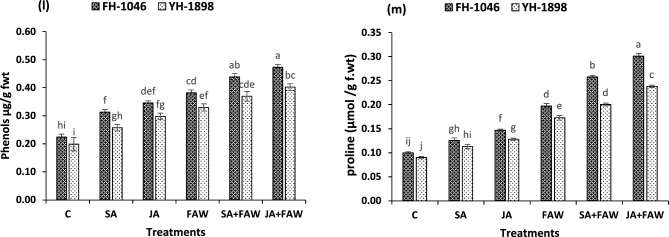
(5 **l**, 5 **m**) Phenol content (µg/g f. wt) and Proline content (µmol/g f. wt) of two maize hybrids after fall armyworm infestation and jasmonic acid and salicylic acid application. Bars (mean ± SE) of same color with similar letters are not statistically different.

Plant responses to herbivore attack are dynamic and intricate, involving various regulatory pathways and biochemical processes. These defenses include some inherent mechanisms to the plant, while others are activated following the plant's recognition of signals linked to insect herbivory^13^. These triggered defensive mechanisms against insect herbivory are controlled by some key phytohormones in which jasmonic acid and salicylic acid play a crucial role^14,15^. These hormones, along with elicitor compounds, can prompt a widespread resistance in plants (Fig. 4). This ability helps plants endure subsequent pest-related harm and has recently become significantly relevant^16,17^. Exogenous application of the phytohormones like jasmonic acid and salicylic acid in modest amounts induces the creation and activation of natural defense systems within the host plants^18,19^. These two phytohormones have been extensively studied for their key role in plant resistance to herbivorous insects, making them an interesting subject of research in agricultural science^20^ (Fig. 5).

**Figure.6:**
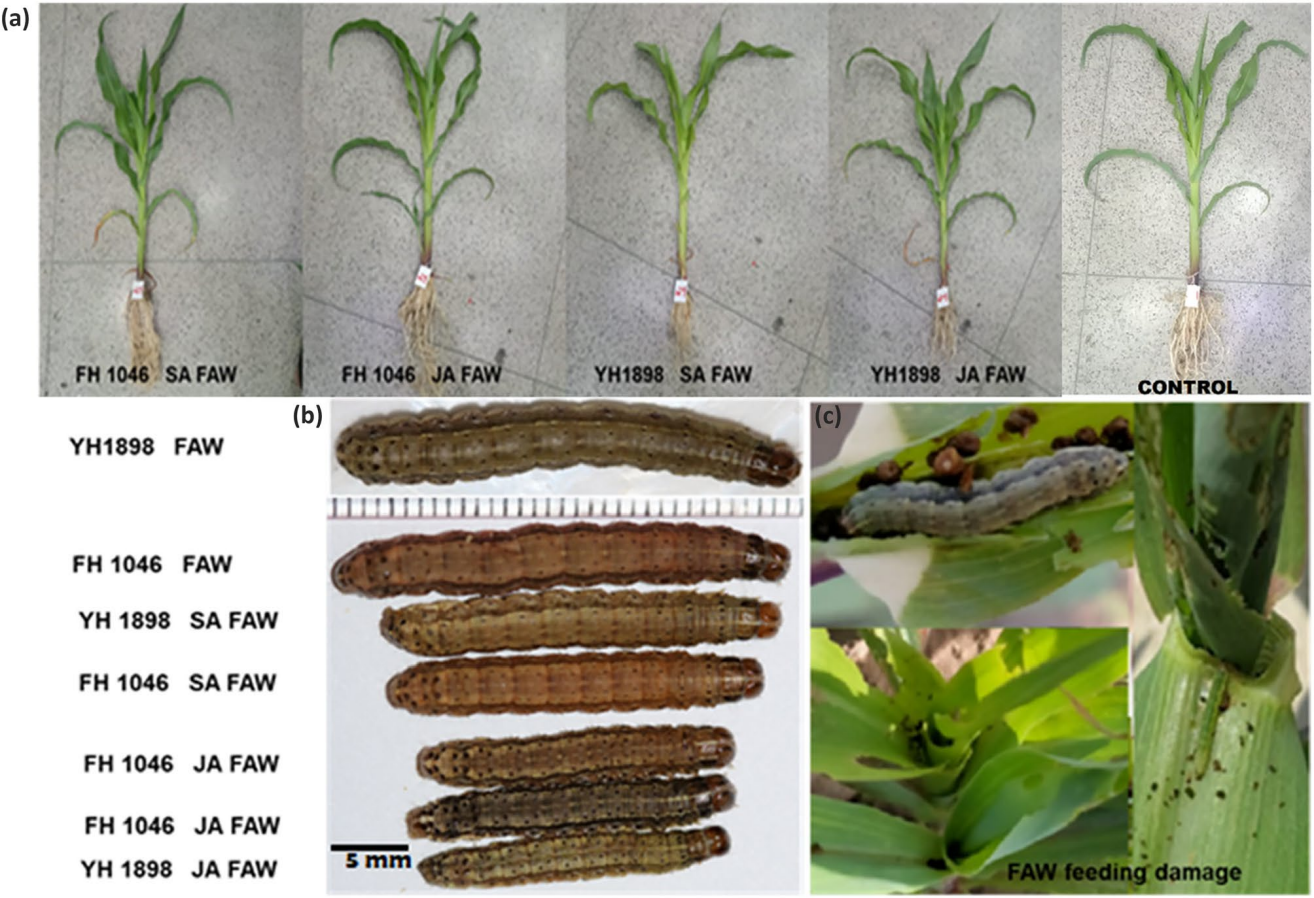
(**A**, **B**, **C**) A. Phenotypic presentation of Zea mays cultivars. FH 1046 treated with SA and JA showed bit increase in size and roots with more mass as compared to YH1898 cultivar. B. After 6 days’ mature stages of FAW larvae on non-treated and treated plants with jasmonic acid and salicylic acid showing significant effect on size and weight of larvae. C. FAW damage pattern on plants.

Although produced in minute quantities, phytohormones are capable of regulating numerous cellular functions in plants. They act as chemical messengers in higher plants to communicate cellular activity. Many studies have demonstrated their significant role in plant protection and stress alleviation ^21^. The role of MeJA and other related hormones has been investigated in a variety of crops, such as wheat, grasses, and sweet potatoes, for their potential in improving abiotic stress tolerance^22^. Salicylic acid (SA), plays a critical role in maintaining plant growth and development and responding to various biotic and abiotic stimuli (Fig. 6). SA activates genes responsible for producing heat shock proteins, antioxidant enzymes, chaperones, and other gene products involved in secondary metabolite metabolism. However, there are little or no reports in literature investigating its role on biotic stress tolerance in plants^23,24^.

Redox homeostasis, the balanced regulation of reactive oxygen species and antioxidants in plant cells, is a critical aspect of plant defense mechanisms against insect herbivory^25^. The delicate balance between ROS production and scavenging influences the activation of various signaling pathways, including those related to defense responses. Alterations in redox homeostasis can have a profound impact on a plant's ability to ward off herbivorous pests^26^. Our research seeks to elucidate the intricate mechanisms by which these phytohormones enhance maize resistance to herbivore attack by modulating redox homeostasis and in turn reviving the plant growth. The findings of this study hold promise not only for advancing our understanding of plant defense responses but also for offering practical strategies to safeguard maize crops against the relentless threat of fall armyworm infestations. This research represents a significant step towards achieving more resilient and productive maize crops, vital for global food security. In the following sections, we will present the methodology, results, and implications of our study, culminating in a comprehensive understanding of the potential of jasmonic acid and salicylic acid in bolstering maize defenses against *Spodoptera frugiperda* infestation.

## Materials and methods

### Plant material

**Figure 7:**
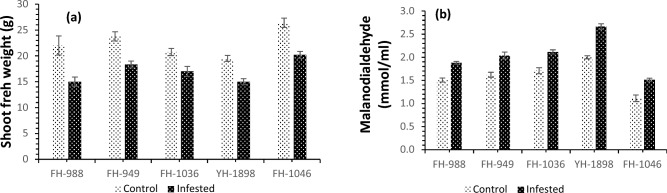
(**A**, **B**) Shoot fresh weight and MDA contents of five maize hybrids from infested and non-infested maize plants.

The seeds of 5 genetically diverse maize hybrids (F-1046, FH-949, YH-1898, FH-1036, FH-988) were obtained from the Ayyub Agricultural Research Institute, Faisalabad, Pakistan. A screening experiment was performed (the results of two parameters including shoot fresh weight and MDA contents are added in this manuscript (Fig. 7. A, B), and two varieties were selected as possible resistant and susceptible varieties on the basis of highest and lowest morphological growth under biotic stress subjected by Fall armyworm infestation following the protocols of^27,28^.

### Ethical statement

It is certified that all the research work conducted in the present experimental study complies with the institutional, national and international guidelines and legislation. All the materials and methods used in this study are approved and registered protocols.

### Treatments

• Control- no treatment/sprayed with water or ethanol (denoted by C).
• Salicylic acid alone (denoted by SA).
• Jasmonic acid alone (denoted by JA).
• Fall armyworm alone (denoted by FAW).
• Salicylic acid + fall armyworm in combination (denoted by SA + FAW).
• Jasmonic acid + fall armyworm in combination (denoted by JA + FAW).

### Chemicals

The chemicals of analytical grade were used for this research including salicylic acid, jasmonic acid (Sigma Aldrich), acetone, trichloroacetic acid, thiobarbutaric acid, potassium phosphate buffer, acidified methanol, sulfosalicylic acid, glacial acetic acid, ninhydrin, aqueous methanol, sodium nitrite, aluminum chloride, sodium hydroxide, Folin-phenol Ciocalteau's reagent, and sodium carbonate.

### Insect culture

FAW Larvae (3rd instar age) were reared in the IGCDB lab (Integrated Genomics Cellular Developmental Biotechnology Laboratory) at the department of Entomology, University of Agriculture Faisalabad, on an artificial diet according to the standard method^29^ under controlled circumstances (27 ± 1 °C, 70 ± 5% RH, and a 10:14 h light: dark photoperiod) until adult emergence. Chickpea flour (175.0 g), yeast (24.0 g), methyl 1–4 hydroxybenzoate (1.50 g), sorbic acid (0.750 g), ascorbic acid (2.350 g), linseed oil (6.0 mL), streptomycin (0.75 g), agar–agar (9.0 g), and double distilled water were used as ingredients to prepare the artificial diet (700.0 mL). After adult emergence, twenty adult pairs were released into transparent plastic jars (15 cm × 15 cm × 30 cm). In order to avoid moths escape, these jars were covered with muslin sheath and a cotton swab dipped with 10% honey solution was added for moth feeding. For oviposition, tissue paper was hanged in plastic jars. Neonates were moved into glass vials containing artificial food after hatching. For the bioassays, second, third, fourth, and fifth instar larvae were reserved.

### Foliar spray solution preparation

A concentration of 1 mM was chosen to induce the activation of SA and JA-dependent defence pathways as our preliminary experiments proved this concentration (out of 3 others tested) was efficient in improving growth and redox homeostasis in maize infested by FAW (data not shown). About 21 mg of JA was dissolved in 1 ml of ethanol to prepare a 1 mM JA solution, which was then diluted in 100 ml of distilled water to make the desired concentration (Hamm et al. 2010). An amount of 0.138 g of salicylic acid was added to one liter of water to prepare 1 mM solution of salicylic acid. Triton X-100 (0.05%) was used as a surfactant here to enhance foliar absorption of the product (Ahmed et al. 2014).

### Sowing and Germination of maize

Pots of 25 cm depth and 15.25 cm diameter were filled with soil  (8 kg each). About 2.50 g of fertilizer was mixed thoroughly in each pot as a recommended dose of N pot^−1^, 1.4 P_2_O_5_ pot^-1^ and 0.9 g K_2_O pot^−1^ (250: 140: 90 kg NPK hectare^-1^, respectively). The seeds were sterilized/treated with 2.5% sodium hypochlorite prior to sowing. After sterilization 12–15 seeds were sown in each pot with equal spacing. The thinning was performed after one week of germination and healthy plants were kept in pots while others were removed. The full strength Hoagland’s solution was provided to plants two times in a week^30^. The irrigation was done on regular basis. The elimination of weeds was performed manually for each pot.

### Infestation procedure

Five larvae were released per plant very carefully onto the plants that were specified for FAW attack. The infested plants were kept in cages to avoid the insects from escaping and to minimize the attack of any other insect pest. Cannibalism was avoided by leaving one insect on top, one on mid section and one on the bottom of plant during infestation.

### Phytohormone application

Foliar applications of salicylic acid and jasmonic acid were performed once before infestation (24 h prior to attack of insects) and then again on 2nd, 4^th^ and 6th day after the infestation of FAW with the interval of 24 h between each treatment. Plants were separately sprayed with each phytohormone at the vegetative stage of growth 3 weeks after germination of maize seedlings). It was made sure that the entire foliage was completely and properly sprayed with the solution. Control plants were either non-sprayed or sprayed with water or ethanol. Phytohormonal applications were performed 4 times to ensure the proper penetration of solution in plants as previously described by^31,32^).

### Collection of insect data (larval weight gain and mortality)

Data regarding insect related parameters was collected from day 1 to 6 of infestation after phytohormonal application. The initial weights (denoted as A) of these larvae were recorded before they were individually introduced to maize leaves treated with JA/SA and untreated (Control) leaves on separate plants. After a 24-h infestation period, we retrieved larvae from the plants, quantified their numbers, and measured their weight to document both insect survival rates and larval weights. These larvae were then carefully reintroduced onto the same plants to continue feeding for an additional period of 48 h. Larval weight was measured by using an electronic weight balance on 2nd, 4th and 6th day of infestation after which larvae were recovered and counted for survival rate or mortality. The live insects were used for carboxyl esterase and Glutathione S-transferase enzyme assay as described below:

### Carboxyl esterase and Glutathione-S- Transferase activity

Larvae fed on treated maize plants with phytohormonal applications were collected. Then 3-h starvation of larvae was performed for the removal of digested material. The protocol of Yasur and Rani (2015) was modified slightly to estimate the activity of carboxyl esterase and Glutathione-S-transferase activity^33^. The grinding of larvae was performed in liquid nitrogen followed by homogenization in homogenization buffer. Homogenization Buffer consisted of 0.1 M phosphate buffer, 1 mM EDTA, 20% glycerol, 1 mM DTT, 1 mM PTU and 1 mM PSF with PH of 7.6. Centrifugation was performed for 15 min, supernatant transferred into new eppendorf tubes and immediately placed on ice to measure the enzyme activity. For measurement of enzyme activity the protocol of (Yang et al., 2004) was used.

### Collection of plant data

After the collection of insect growth data, fresh leaf samples for determination of chlorophyll contents and relative water content were also detached from each treated and non-treated plant. After this, the plants were harvested and samples were immediately frozen and stored at − 80 °C until further use. The physiological analysis were performed on 10 day frozen samples.

### Growth related parameters

A meter rod was used to measure the lengths of the shoot, roots and plant height. Following the dissection of roots and shoots, an electric balance was used to determine the fresh and dry weights of each sample. The samples were sun-dried for three days firstly and then oven-dried for a constant weight at 75 °C to determine the dry weight of the roots and shoots.

### Photosynthetic pigments

The procedure by Arnon (1949) was used for the determination of chlorophyll a, b, carotenoids and total chlorophyll contents^34^. 0.1 g fresh leaf was chopped and placed in plastic bottles containing 5 ml of 80% acetone solution. These samples were placed over night at 4 °C. After that centrifugation was performed at 10000 rpm for 5 min. Absorbance of samples for chlorophyll a, b and carotenoids was measured at 645 nm and 663 nm, 470 nm wavelength on U-2900 HITACHI spectrophotometer.

### Relative water content

The protocol of Barr and Weatherley (1962) was followed for measurement of relative water content^35^. The fresh weight of a flag leaf was determined with the use of an electronic scale. Following a 24-h soak in distillate water, the weight of these leaves was again determined in grams using an electronic balance. Then leaves were oven dried at 70 °C and dry weight was measured once again after which the values were computed in the formula to find the water content of samples.

### Superoxide dismutase and peroxidase activity

Using the method outlined by Chance and Maehly (1955), peroxidase enzyme was measured^36^.  The supernatant was extracted using phosphate buffer with a pH of 7.8. About 100ul of supernatant, 100ul of guaicol, 1 ml of buffer, and 100ul of hydrogen peroxide were added  to a cuvette.  The absorbance was measured at 470 nm after 0, 20, 40, 60 and 80 seconds. Protocol of Giannopolities and Ries (1977) was followed for measurement of SOD activity. The 3 ml final volume of reaction mixture used for this purpose consisted of enzyme extract 50 µL, NBT (50 µl), Triton X (100 µl), methionine (100 µl), distilled water (400 µl), riboflavin (50 µl) and phosphate buffer (250 µl), having pH 7.6. Test tubes filled with reaction mixture were exposed to fluorescent light for 15 min at room temperature. After 15 min reading was measured at 560 nm spectrophotometer.

### Polyphenol oxidase activity

The Mayer and Harel (1979) technique for estimating polyphenol oxidase activity was used with a few modifications^37^. Enzyme source 0.1 ml and 0.1 ml of substrate (0.05 M catechol) were added to 2.9 ml of 0.1 M sodium phosphate buffer (pH 6.8). At 30-s intervals, the absorbance was measured at 420 nm for 3 min. The measure of enzyme activity was in IU g^-1^ FW. A change in absorbance of 0.1 units per minute under assay conditions was used to define one unit of PPO. One PPO unit was defined as a 0.1 unit per minute change in absorbance.

### MDA (Malondialdehyde)

The determination of MDA content was performed by using the procedure of Heath and packer (1968)^38^. A 0.5 g of leaf sample was homogenized in 3 ml of trichloroacetic acid and centrifugation was performed at 11,500 rpm for 15 min. About 2 ml of supernatant was separated and mixed with 4 ml of thiobarbutaric acid and heated at 95C for 30 min. Again, centrifugation was performed at 11500 rpm for 10 min. Absorbance measurement was performed at 532 and 600 nm. Following formula was used for MDA contents computation.

MDA = (A532-A600)/ɛ Here ɛ is the extinction coefficient.

### H_2_O_2_ (Hydrogen peroxide)

Protocol of Velikeva et al. (2000) was followed to measure H_2_O_2_ contents. A 0.5 g fresh leaf sample was homogenized in prechilled pestle and mortar by using 0.5 ml of 0.1% TCA. Centrifugation was performed at 12,000 rpm for 15 min. An amount of 500ul supernatant, 500ul of potassium phosphate buffer and 1000ul of 1 molar KI were mixed and absorbance was checked at 390 nm.

### Phenolic contents

Leaf total phenolics were measured using the Julkenen-Titto method (1985)^39^.  Fresh leaf sample (0.5g) was homogenized in an 80% acetone solution. After that, the homogenized leaf material was centrifuged at 10,000 × g for 10 min to separate the supernatant. Following that, a reaction between an aliquot (100 l) of the supernatant and 1 ml of Folin-phenol Ciocalteau's reagent and 2.0 ml of distilled water was carried out. After adding 5.0 ml of a 20% Na2CO3 solution to the mixture, the final volume was increased to 10 ml by adding distilled water. A UV–Visible spectrophotometer (IRMECO U2020) was used to measure the OD at 750 nm after vigorous mixing (GmbH, Germany).

### Proline content

In 5 ml of 3 percent sulfosalicylic acid, 0.05 g of plant material was homogenized according the methodology given by Bates et al. (1973). For complete extraction this mixture was placed for 3 h. After that centrifugation was performed at 1500 rpm for 10 min. And then 2 ml supernatant, 2 ml glacial acetic acid and 2 ml ninhydrin were added in test tubes and these tubes were placed in water bath at 100C for 60 min. These test tubes were placed in an ice bath to stop the reaction. 4 ml toluene added in the test tubes and vortexed them until two layers formed and a ring.

### SA/JA mediated Gene expression Study

#### RNA extraction

Total RNA from leaves of healthy and infected maize cultivars was isolated by the TriZol LS-Reagent® method (Invitrogen- Carlsbad, MI, USA). Briefly, 0.2 g to 0.5 g of frozen, leaves were ground in liquid nitrogen. The powder was mixed well with 1 ml of TriZol LS-Reagent® and extraction was done following the manufacturer’s protocol. The nucleic acids were dissolved in 15 μl sterile deionized water. RNAs (8 μg to12 μg) from leaves were treated with 5 units of RQ1 RNase- Free DNase (Promega, Madison, WI, USA) at 37 °C for 1 h in the presence of 1X reaction buffer (Promega, Madison, WI, USA) in a final volume of 50 μl following the manufacturer’s instruction. The nucleic acid concentration was determined by spectrophotometry at λ = 260 nm.

#### Reverse transcription Complementary DNA (cDNA) and Real Time PCR

The cDNA was synthesized from 1 μg of RQ1-DNase-treated RNA extract from leaves of healthy, and infected plants by using 5 μM oligodT18 primer or specific reverse primers and 200 units of Superscript® II Reverse Transcriptase (Invitrogen, Carlsbad, CA, USA) in a 25 μl reaction mixture containing 10 mM DTT, 20 μM dNTPs and 40 units of RNase Out™ (Invitrogen). The reaction was performed according to the recommended protocol^40^ except that the mixture (RNA + oligodT18 or specific primer) was heat-denatured at 65 °C for 5 min before adding the reverse transcriptase and the other components. Controls, without reverse transcriptase, were used to verify the efficiency of the DNase treatment. For real time PCR, each reaction tube contained 1 × SYBR Green Fluorescein Mix (Applied Biosystems), primers at 250 nm each and 1μL cDNA in a final volume of 25μL. The thermal cycling programme was as follows: 95 °C for 15 min, 45 steps each of 20 s at 95 °C, 40 s at the annealing temperature (which was adjusted according to the primer pair used (Table 3) and 40 s at 72 °C. The cycling programme was followed by a melting-curve programme: 30 s at 95 °C, 10 s at 60 °C and a melting step from 60 °C to 100 °C (step of 0·5 °C every 10 s) for 80 cycles, and a final step of 10 min at 72 °C. All RT-PCR analyses were carried out in triplicate as described above. Efficiencies of all primer pairs were determined using tenfold dilutions of the RT-PCR product (undiluted, 1/10, 1/100, 1/1000, 1/10,000) using CFX 96 Real Time BioRad software and were found to range from 95 to 110%. NThe results were analyzed using relative gene expression (RGE)^41^, according to the formula presented in^42^^.^

**Table 3:** SA/JA mediated forward and reverse genes primers used in qRT-PCR.

Primers names	Sequences (5'-3')	TM (℃)
SPI-F	AAGAGATTGGTGCTCTGCCC	60.03
SPI-R	CGCGAAATTTCCGTCCCATC	60.00
WRKY28-F	TTGGCCGTCAGGACGAAAG	60.03
WRKY28-R	CCGTACTTCCGCCATTGGTA	59.82
ICS-F	GCATCATCCGCATCGAGGT	60.03
ICS-R	CGCGCCAATCAGCTAGAGAA	60.53
NPR1-F	CCCTGAGCATGACAAGAGGG	60.11
NPR1-R	CACCTTGGGCTCACAGTAGG	60.04
PAL-F	GTCGTCCACCTACATCGTGG	60.18
PAL-R	ACCTGGGTCACGGTGTTCTT	61.34
PR1-F	TCGCACATCAAGGTGGAGC	60.02
PR1-R	ATGGTTTAGTTGTAGGCGTCGG	60.00
LOX5-F	TCGCGTCTACCGTTACGACTACTA	59.80
LOX5-R	TTCAGGTTCAGCAGGAAAAGC	59.98
TPS10-F	ATCTCACCCTTCAAACCCC	60.05
TPS10-R	TAACCTCTTTCAACTCCTCAC	60.00
BBT12-F	TGGGACTGCTGCGACTTCG	60.03
BBT12-R	GCATCGGTAGCCAGGAGGG	59.98
ACTIN-F	CGGCAGCCTCCATACCAA	60.02
ACTIN-R	GCCAAGAACAGCTCCTCA	60.00

#### Plant damage Assay

Plant damage was carefully observed by detecting webs on leaves.

### Statistical analyses

The experiment was conducted in a completely randomized design with 3 replications. The replication data was arranged and means and standard deviation were calculated. Analysis of variance (ANOVA) was used to examine all data in order to compare treatments among genotypes and within a genotype under split plot design. The results of genes expression are presented as the means ± standard deviation (SD). Student’s t-test was used to analyze the significant differential analysis of expression. One-way ANOVA followed Duncan’s test was used for comparison of growth inhibition rate among different treatments. All statistical analyses were conducted using SPSS software v20. P-values.

## Results

### Screening experiment

The results of the pilot experiment (Fig. 7) showed FAW attack increased the MDA content and reduced the shoot fresh weight of all 5 cultivars, however, the increase and decrease was more evident in the susceptible cultivar YH-1898. On the other hand, the cultivar FH-1046 showed resistance against the FAW infestation and maintained its growth and biomass due to lower oxidative stress (as shown by low MDA content in Fig. 7).

### Larval survival, weight and plant damage

**Figure.8:**
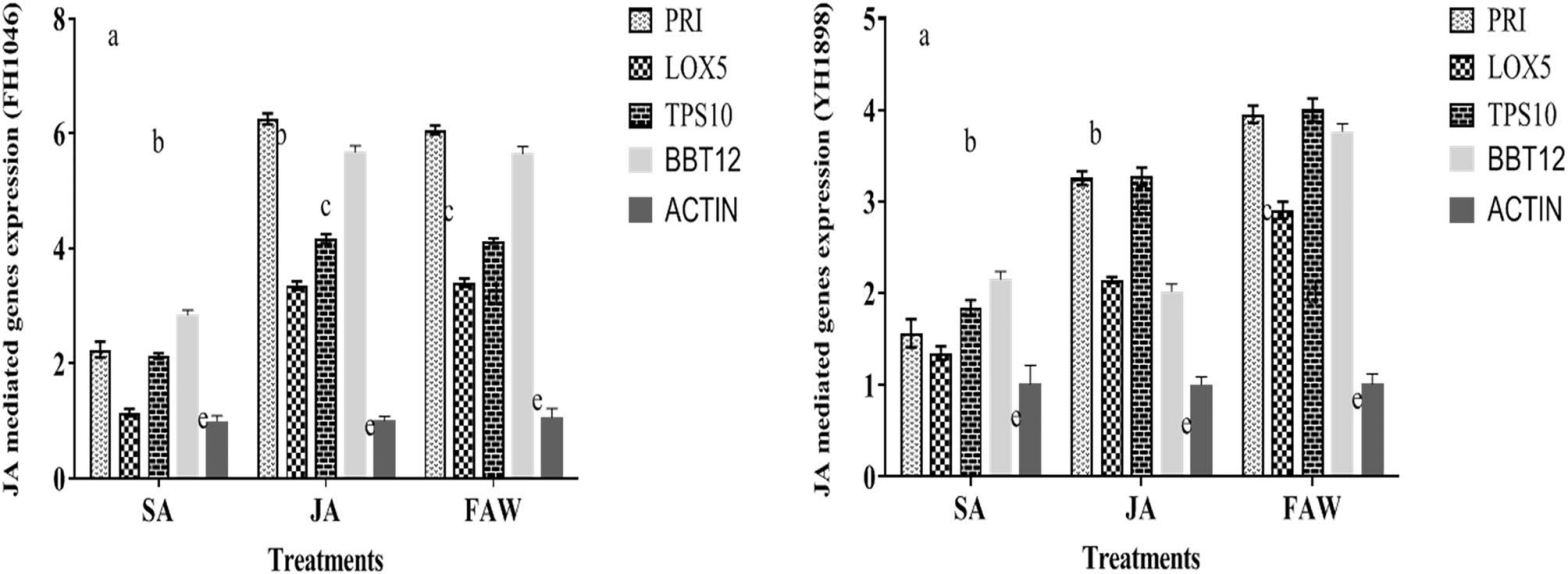
Relative expression of JA mediated genes in response to foliar application of phytohormones (SA and JA) and FAW infestation. Actin was used as control gene in real time PCR.

**Figure.9:**
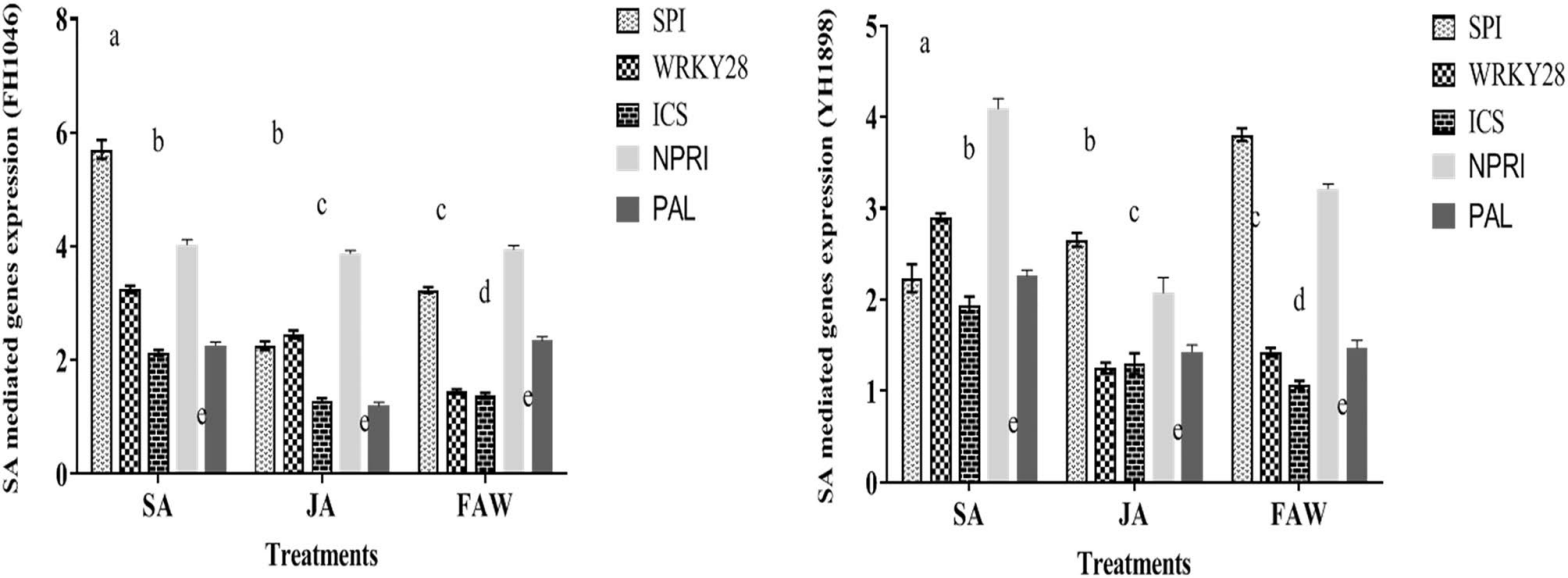
Relative expression of SA mediated genes in response to foliar application of phytohormones (SA and JA) and FAW infestation. Actin was used as control gene in real time PCR.

Physical plant damage caused by armyworm was more obvious in YH-1898 than FH-1046 in both screening and main experiment. It showed that YH-1898 is more vulnerable to armyworm attack in comparison to FH-1046. However, leaf damage was low in plants treated with JA + FAW followed by SA + FAW in FH-1046 compared to YH-1898 (Fig. 8). Highest damage was observed in the control set (plants without phytohormone treatment). Larval survival was significantly lower in FH-1046 in JA + FAW, SA + FAW respectively compared to FAW alone (Fig. 9). The weights of the larvae retrieved at 6th day were lower in FH-1046 than YH-1046 in JA + FAW and SA + FAW compared to FAW as shown in Figs. 1 and 6b.

### Photosynthetic pigments and Relative Water Content (RWC)

Results revealed that FAW attack significantly reduced photosynthetic pigment and RWC in maize leaves. However, JA application on infested plants significantly improved their photosynthetic pigments and relative water content. Highest values for these attributes were found in 1 mM JA treated plants followed by 1 mM SA treated plants. Chlorophyll a improved from (0.155–0.161 mg/g fwt.) in FH-1046 and (0.131–0.150 mg/g fwt.) in YH-1898 after JA spray. Chlorophyll b improved from 0.146–0.182 mg/g fwt. in FH-1046 and 0.109–0.181 mg/g fwt. in YH-1898 after JA spray. Similarly, carotenoid contents increased from 0.025–0.039 mg/g fwt.) in FH-1046 and 0.015- 0.029 mg/g fwt. in YH-1898 after JA spray. Relative water contents increased from 66.5–70.8% in FH-1046 and 62.6–68% in YH-1898 after JA spray. FH-1046 showed highest amount of Chlorophyll a (*p* ≤ 0.01), b (*p* ≤ 0.01) and carotenoid contents with (*p* ≤ 0.001) in comparison to YH-1898 as shown in Fig. 2.

### SOD and POD activity

Results revealed that antioxidant activity of SOD was lowest in control/no spray conditions (7.132 ± 0.004 mg^−1^ protein) in FH-1046 and (5.59 ± 0.176 mg^−1^ 245 protein) in YH-1898. While SOD activity in infested plants was higher than control (12.22 ± 0.080 mg^-1^ protein) in FH-1046 and (11.10 ± 0.027 mg^−1^ protein) in YH-1898. This activity was augmented after JA application (16.70 ± 0.138 mg^−1^ protein to 14.77 ± 0.052 mg^−1^ protein) in FH-1046. Similarly, lowest POD activity (0.64 ± 0.10 IUg^−1^FWT) was observed in FH-1046 and (0.35 ± 0.06 IUg^−1^FWT) in YH-1898 in control conditions and highest (2.47 ± 0.05 to 3.45 ± 0.10 IUg^-1^FWT) in FH-1046 and (1.92 ± 0.08 to 2.74 ± 0.06 IUg^−1^FWT) in YH-1898 when JA was applied along with FAW infestation. It was concluded that highest SOD (*p* ≤ 0.01) and POD activity (*p* ≥ 0.01) was found in plants with JA + FAW followed by SA + FAW, FAW, JA, SA and C. But FH-1046 had higher level of SOD and POD activity compared to YH-1898 as shown in Fig. 3.

### Polyphenol oxidase activity

Results revealed that PPO activity was lowest in control plants (0.05 ± 0.001 IU g^−1^ FWT) in FH-1046 and (0.04 ± 0.001 IU g^−1^ FWT) in YH-1898. While infested plants showed higher activity of PPO activity after JA application from 0.14 ± 0.022 IU g^−1^FWT to 0.33 ± 0.002 IU g^−1^ FWT in FH-1046 and 0.13 ± 0.002 to 0.32 ± 0.003 in YH-1898. Results indicated that PPO activity (*p* ≥ 0.01) was highest in plants with JA + FAW followed by SA + FAW, JA, SA, FAW and C. But FH-1046 had higher level of PPO contents compared to YH-1898 as shown in (Fig. 3).

### Oxidative stress indicators

Results revealed that MDA and H_2_O_2_ were significantly high in FAW infested plants as compared to control. MDA contents reduced with JA application in infested plants from 1.6–1.4 µmol/g f. wt in FH-1046 and 1.8–1.5 µmol/g f. wt in YH-1898. H_2_O_2_ contents were also highest in FAW infested plants which were reduced with JA treatment from (2.25–1.96 µmol/g f. wt) in FH-1046 and (3.25–2.96 µmol/g f. wt) in YH-1898. MDA (*p* ≥ 0.01) and H_2_O^2^ (*p* < 0.01) were found highest in FAW followed by SA + FAW, JA + FAW, C, SA, JA. But YH-1898 had higher level of MDA and H_2_O_2_ compared to FH-1046 as shown in (Fig. 4).

### Metabolites production

Results revealed that metabolites production including proline and phenolics was lowest in control plants in both hybrids. Phenolics contents increased when FAW attacked the plants. This increase was augmented with JA application in infested plants from 0.38 to 0.47 μg/g f. wt in FH-1046 and 0.33–0.40 μg/g f. wt in YH-1898. Phenolic contents were highest (*p* ≥ 0.01) in plants with JA + FAW followed by SA + FAW, FAW, JA, SA and C. But FH-1046 had higher level of phenolic contents compared to YH-1898. Proline contents were also significantly high (*p* ≤ 0.01) in FAW infested plants (Table 4). These levels were more pronounced when JA and SA were applied to the plants (0.20–0.30 μmol/g f. wt) in FH-1046 and (0.17–0.24 μmol/g f. wt) in YH-1898 as shown in (Fig. 5).

**Table 4:** Relative expression of JA and SA mediated genes in response to foliar application of phytohormones (SA and JA) and FAW infestation.

Genes names	FH1046			YH1898		
	SA	JA	FAW	SA	JA	FAW
SA mediated genes expression (fold times)						
SPI	5.70a	2.25e	3.23b	2.30e	2.65g	3.80d
WRKY28	3.24b	2.44e	1.43f.	2.90g	1.24f.	1.42f.
ICS	2.12c	1.29f.	1.37f.	1.94c	1.30f.	1.00 h
NPR1	4.01d	3.87d	3.94d	4.09d	2.07c	3.21b
PAL	2.26e	1.19f.	2.34e	2.26e	1.43f.	1.47f.
JA mediated genes expression						
PR1	2.30b	6.25a	6.03a	1.50f.	3.25b	3.95d
LOX5	1.14f.	3.34b	3.40b	1.34f.	2.14c	2.90g
TPS10	2.12c	4.16d	4.12d	1.84c	3.26b	4.00d
BBT12	2.85 g	5.67a	5.64a	2.15c	2.00c	3.76d
ACTIN	1.00 h	1.00 h	1.00 h	1.00 h	1.00 h	1.00 h

Actin was used as control gene in real time PCR.

### Morphological growth

The results revealed that JA and SA treated plants showed highest morphological growth while FAW infested plants showed lowest growth in both FH-1046 and YH-1898 (Table 1 and Fig. 6a). FH-1046 showed significantly highest morphological growth with (*p* ≤ 0.001) in comparison to YH-1898. Morphological growth increased in infested plants after JA application. Plant height increased from 62-68 cm in FH-1046 and 53–58 cm in YH-1898, Similar results were obtained in case of root length (30–34 cm) and 24- 29 cm, shoot length (51–53 cm) and 44–50 cm, shoot fresh weight (44.94–50 g) and (39.2–47.32 g), in FH-1046 and YH-1898 respectively. The recorded data makes it evident that JA application followed by FAW attack helped the maize plants defend themselves and revived their growth significantly as denoted in (Table 1 and Fig. 6b).

### Detoxification enzymes activity

Investigation of carboxyl esterase and glutathione-s-transferase (GST) activity in *S. frugiperda* against phytohormonal application revealed that carboxyl esterase and GST enzyme activity was reduced with phytohormonal application. Our results indicated that carboxyl esterase activity was significantly high (*p* ≤ 0.001) in FAW infested plants which was depreciated with JA treatment in both varieties. Glutathione-s-transferase significantly (*p* ≤ 0.001) decreased with JA treatment in FH-1046 and in YH-1898 as shown in Table 2.

### Relative genes expression with JA-SA-FAW treated maize

The expression of SA mediated genes in two selected cultivars of maize (FH1046 and YH1898) were significantly different (Table. 4. and Fig. 9). The expression of SP1 was found to be enhanced 5.50 fold as compared to 2.30 on SA application in FH1046 and YH1898 respectively. There was also significant difference of SP1 expression on JA and FAW infestation in both cultivars. Similarly, the expression level of WRKY28, NPR1 was higher in FH1046 maize cultivar on SA application and FAW infestation as compared to YH1898. The expression of SA synthesis enzymes such as ICS and PAL was found higher on SA application which indicated that SA was involved in induction of these genes. Similar mechanism of defensive and vital synthesis genes or markers was found for JA mediated genes as shown in (Fig. 8). For example, JA responsive genes as PR1, and JA synthesis enzyme Lox5 was expressed 6.25 fold and 6.03 fold in FH1046 maize cultivar on JA application and FAW infestation as compared to control Actin gene. The other responsive genes TPS10 and BBt12 was found activated much as compared to SA application in both maize cultivars. Over all, JA mediated genes were over expressed on JA application and FAW infestation as compared to SA application in both maize cultivars. The difference in expression level may be associated with different maize cultivars as both showing different phenotypic and metabolic secondary metabolites level on the application of different phytohormones and FAW infestation (P < 0.05) (Table 4.). Due to differential expression of responsive genes/SA/JA synthesizing enzymes in both maize cultivars, the mortality and weight gain/loss of FAW on both cultivars was observed. The JA mediated defense path way seems to be stronger in FH1046 maize cultivar as compared YH1898.

## Discussion

Induced resistance in plants is regarded as an admirable crop defense strategy with minimal adverse impacts^43,44^. Innate plant defense system involves several morphological, biochemical, and molecular processes to counteract the herbivore attack. Majority of the plant defense reactions against insects are triggered by signal-transduction pathways mediated by phytohormones like JA, SA and ethylene^24^. Phytohormones play a pivotal role, orchestrating defense mechanisms that help plants to adapt and counteract herbivore damage. Among these phytohormones, Jasmonic Acid (JA) and Salicylic Acid (SA) have gained prominence as essential modulators of defense responses in plants against external stimuli^45^. The external treatment of these phytohormones (JA and SA) stimulates both direct and indirect forms of resistance by influencing vital signaling paths such as the octadecanoid and hexadecanoid routes^46,47^. JA induced octadecanoid, SA induced redox pathway and production of various defensive compounds are involved in resistance induction in plants against insect herbivory^48,49^.

Morphological traits play a pivotal role in a plant's defense mechanism against biotic stressors, showcasing an intricate adaptation to environmental challenges. Plant height, root-shoot lengths, and biomass allocation are crucial parameters that contribute significantly to their resilience^23,50^. In the present investigation, depreciation in various morphological attributes was recorded in plants infested with fall armyworm. Our findings corroborate previous studies demonstrating the positive impact of foliar application of jasmonic acid on wheat growth parameters under aphid infestation and common bean plants growth improvement under spider mite infestation^51–54^. The observed increase in plant height, root and shoot fresh weight, as well as lengths of maize plants in the present study, aligns with established knowledge regarding the role of jasmonic acid in promoting plant development. This reaffirms the efficacy of utilizing jasmonic acid as a viable strategy to enhance maize growth, suggesting its potential application in agricultural practices aimed at improving crop yield and vigor. The consistency of our results with prior research underscores the robustness and reliability of employing jasmonic acid as a means to augment plant growth parameters in maize.

Photosynthetic pigments and flag leaf area in plants are not just vibrant hues; they're nature's defenders. They function as vigilant protectors, intercepting excessive light and combating oxidative stress caused by herbivory^55^. Acting as conductors, they coordinate a plant-wide defense, signaling nearby leaves to fortify against grazing. Furthermore, these pigments possess the power to render leaves unappealing or even harmful to herbivores, ensuring the plant's resilience in the face of feeding pressures^56,57^. Carotenoids represent a class of lipophilic compounds, derived from isoprenoids, synthesized across various organisms^58^. Within plants, they serve as vital photosynthetic pigments, crucial for mitigating excessive light energy and scavenging reactive oxygen species (ROS) produced during photosynthesis^59^. The levels of carotenoids can be influenced by biotic stresses like herbivory and pathogen infections. Intriguingly, recent findings suggest that additional carotenoids and/or apocarotenoids play a role in triggering retrograde signals, which in turn regulate the expression of specific nuclear genes, thereby profoundly impacting plant developmental pathways and alternative responses to stress^60–63^. Moreover, the presence of high concentrations of carotenoids has been associated with diminished efficacy of engineered anti-herbivore Bt defenses, as they facilitate herbivore detoxification through ROS scavenging mechanisms^64^.

The impact of biotic stress on photosynthetic pigments in plants has been extensively documented in prior studies^65–67^. Our findings align with these research studies, as we also observed a notable reduction in chlorophyll a, b, carotenoid contents, and flag leaf area in maize leaves affected by FAW infestation. Intriguingly, our experimentation with jasmonic acid treatment showcased a significant mitigation of these losses, demonstrating an enhancement in photosynthetic pigments and leaf area even in infested plants. This observation reveals the outcomes documented in other experiments^51–53, 68, 69^, further bolstering the potential efficacy of phytohormonal treatments in counteracting the adverse effects of biotic stress on plant physiology.

Relative water content is amount of the water content in plant tissues relative to their fully hydrated state. It offers valuable information regarding water status and hydration level of plants. RWC plays a valuable role in plant growth, development, and responses to various stresses, including salt stress and insect attack^70^. It reflects the capability of plants to absorb, hold, and distribute water for proper functioning. Maintaining an adequate RWC is vital for cell turgidity, metabolic processes, nutrient uptake, and overall plant growth^71^.

Previous studies, as delineated^51^, consistently highlight a decline in relative water content during biotic stress across various crop species. Notably, plants treated with jasmonic acid exhibited the highest relative water content, even surpassing levels observed in infested plants. Conversely, infested plants consistently demonstrated the lowest levels of relative water content. Furthermore, the application of salicylic acid exhibited a remarkable capacity to enhance the relative water content in stressed plants. Complementing these findings, earlier investigations,^72^ which supports the notion of increased relative water content following foliar application of JA confirming the ability of phytohormones to possibly revive plant growth and recovery post insect damage.

It is a well-documented phenomenon that an upsurge in antioxidant enzyme activity serves as a response to stress conditions^73^. Antioxidants in plants are crucial defenses against damage caused by herbivores. They help neutralize reactive oxygen species generated during herbivory, minimizing cellular damage. Additionally, these antioxidants may influence herbivore behavior by altering plant tissue quality. Understanding this interplay is key to deciphering plant defense mechanisms and exploring pest management strategies^74,75^. SOD (superoxide dismutase) and POD (peroxidase) enzyme activity is thought to be a plant's immediate defense mechanism against abiotic stress and herbivory that involves cell wall strengthening^76–78^. Foliar spray with MeJA improved growth and yield by improving photosynthetic pigments, antioxidant activity, endogenous JA and reducing oxidation stress in rice^47,79^. Salicylic acid also oversees the functioning of several enzymes like POD, PPO, SOD and PAL. These enzymes constitute key elements of the induced defense response in plants against both biotic and abiotic pressures^80^.

In our current investigation, a notable elevation in antioxidant activity was noted in plants treated with a combination of JA and FAW, closely followed by those treated with SA and FAW. Remarkably, within the studied varieties, the resistant variety FH-1046 exhibited pronounced antioxidant activity in comparison to YH-1898. This underscores the pivotal role of SOD and POD activity as integral components in the rapid response mechanism against insect-induced damage, aligning with findings from prior studies^54,81–85^.

Polyphenol oxidase (PPO) activity within plants assumes a paramount role when they face biotic stresses. It's a crucial defender that helps plants fight off these threats by triggering important chemical reactions. When PPO gets active, it strengthens the plant's defenses by building tougher cell walls and producing substances that keep invaders at bay^73,86^. Under the duress of biotic stressors, the surge in polyphenol oxidase (PPO) activity serves as a pivotal defense mechanism within plant systems.

Our investigation reveals a compelling augmentation in PPO activity amid stress imposition. A notable elevation in PPO activity was discerned in plants treated with combinations of JA and FAW, as well as SA and FAW, specifically in FH-1046. Interestingly, YH-1898 exhibited comparatively lower PPO activity across all treatments in contrast to the robust response observed in FH-1046. In the present study, the increase in PPO activity was corroborated by a noteworthy escalation following the application of jasmonic acid treatment. This finding echoes the resonance of prior studies, aligning harmoniously with established research. Previous studies have consistently reported the role of JA application in augmenting PPO activity amidst insect attacks^73,87, 88^. This induction of enzymes in response to insect onslaught plays a crucial role in metabolite production and activation of the phenylpropanoid pathway, thus fortifying the plant's resilience against herbivory, as elucidated in studies^81^. The heightened PPO activity observed during stress instances signifies an adaptive response, showcasing the intricate orchestration of biochemical pathways in fortifying plant resilience against biotic challenges^89^. Furthermore, the paradigmatic correlation between stress-induced escalation and the further amplification due to jasmonic acid treatment accentuates the integral role of PPO in the intricate tapestry of plant defense mechanisms, delineating a pivotal facet in understanding and harnessing plant stress responses for agricultural enhancement^85^. During instances of biotic stress in plants, the intricate interplay of signaling pathways unfolds to combat external pressures.

Malondialdehyde (MDA) emerges as a consequential biomarker, reflecting oxidative stress levels^90,91^. Concurrently, hydrogen peroxide (H_2_O_2_) assumes the role of a secondary messenger, orchestrating pivotal signaling cascades. The onset of stress triggers the activation of specific enzymes like NADPH oxidases, catalyzing H_2_O_2_ production, which, in turn, transmits stress signals to activate defense mechanisms ^92^. However, this signaling pathway demands a delicate equilibrium; while imperative for stress resilience, excessive MDA and H2O2 can potentially impair cellular function. In our study plants infested with FAW (fall armyworm) unveiled heightened levels of MDA and H2O2, affirming their roles in initiating reactive oxygen species (ROS) production, pivotal for the synthesis of defensive compounds. Notably, the application of phytohormones mitigated these stress-induced elevations, showcasing their protective role against biotic stress. Among these, Jasmonic acid exhibited the most pronounced reduction in MDA and H_2_O_2_ levels, underscoring its potential as a robust defense enhancer in stressed plants as document earlier^53,68^.

Osmolytes accumulation plays a major role in osmotic adjustments under stressful environmental conditions^93^. Proline accumulation is observed in plants as a counter response towards various abiotic stresses and pathogen attacks^94,95^. Proline's role in plant response to drought stress is well-documented. It acts as an osmoprotectant, helping to maintain cellular hydration and structure during water scarcity. However, its function in maize resistance to pests is a bit more nuanced. Proline's involvement in pest resistance in plants is linked to its role in signaling pathways. Elevated proline levels have been associated with enhanced defense mechanisms against certain pests^96^. When plants are attacked by pests, they trigger various defense responses, including the accumulation of proline. This accumulation serves as a signal to activate defense-related genes and pathways, bolstering the plant's ability to withstand pest attacks. Higher proline levels might indicate a plant's active response to pest attack, reflecting its defense mechanisms in action^97,98^. Maize employs various defense strategies against pests, including physical barriers, chemical compounds, and signaling pathways beyond proline accumulation. However, it's essential to note that proline's role in pest resistance is just one aspect of a complex defense system in plants. Plants subjected to JA + FAW treatment exhibited elevated levels of proline content, surpassing those treated with SA + FAW. This highlights the role of phytohormones in augmenting the accumulation of proline during instances of insect attack^85^.

Phenol accumulation has adverse impacts on insect development and feeding which is a common response in plants toward herbivory^99^. Phenolic compounds, including flavonoids and tannins, are secondary metabolites in plants that play a significant role in plant defense against biotic stressors^100^. These compounds often accumulate in response to herbivore attack, acting as both physical and chemical barriers^101,102^. These compounds often accumulate in response to herbivore attack, acting as both physical and chemical barriers^101,102^ Phenolics can deter herbivores by interfering with their digestion making plant tissues less palatable. Actually phenolics form complexes with proteins, thereby decreasing protein digestion and ultimately making amino acids unavailable for the insect’s nutrition^102^.

Phenolics also have antioxidant properties and can help in mitigating oxidative stress induced by herbivore feeding^50,103^. Phenol accumulation was boosted in infested plants after JA application revealing harmony with previous study^83^.

Insects rely exclusively on innate immunity, which encompasses both humoral and cellular components. Cellular immune system and humoral immunity involve diverse processes like nodulation, clotting, phagocytosis, melanin synthesis etc.^104^. One tactic employed by insects to neutralize plant defensive compounds involves production of detoxification enzymes. Typically, there are three main types of detoxifying enzymes which deal with plant allelochemicals and synthetic pesticides; cytochrome P450, monooxygenases and carboxylesterases^104–106^. These detoxifying enzymes are responsible for various mechanisms including reduction, hydrolysis, oxidation, and conjugation of plant allelochemicals and pesticides^107,108^. Carboxyl esterase and GST (Glutathione S transferase) both perform specific roles as antioxidants and are involved in the defense system of insects against unfavorable environmental conditions^109^.

The activities of the enzyme glutathione S transferase (GST) experienced a notable decline (*p* < 0.01) in larvae reared on maize leaves treated with phytohormones when compared to the control group. The impact of the 1 mM JA treatment was significant and more pronounced than other treatments. Similarly, when response was observed regarding carboxylesterase activity in fall armyworms, a significant reduction was revealed in insects feeding on jasmonic acid and salicylic acid treated plants in comparison to control. Depreciation in detoxification enzyme activities in our study was in harmony with earlier studies^106^. Our findings are also consistent with prior experiment^110^ as they also observed reduced carboxylesterase activity in adult *Monolepta hieroglyphica*. Similar findings were reported when cotton bollworms were fed on MeJA-treated cotton plants^111^. Moreover, in our study, we observed a parallel outcome to previous research where the exogenous application of salicylic acid (SA) on tomato plants inhibited detoxifying enzymes activity in *Tetranychus urticae* reported^112^. Inhibition of insect digestive enzyme activities renders them more susceptible to plant defenses, thereby curtailing insect growth and development. The herbivore's growth and development are substantially influenced upon consuming induced plants^113^. Insects feeding on these induced plants encounter increased mortality rates, delayed development, and lower body weight compared to those feeding on control plants. This might be attributed to the presence of heightened toxic elements like protease inhibitors (PIs) or other harmful metabolites^113,114^. Plants possess inducible defense mechanisms that serve as inherent weapons against herbivores. The activation of these defense systems and the synthesis of protective compounds within plants can be heightened by externally applying substances called phytohormones, such as jasmonic acid and salicylic acid. This augmentation results in an increased production of various metabolic compounds^18,115–117^. The interactions between JA-induced plants and herbivores have long been a focal point of research. Both jasmonic acid and salicylic acid prompt the production of harmful secondary compounds which hinder nutrition within plants. These substances have the effect of curtailing the development of larvae, resulting in discouragement of adults from laying eggs^118,119^. As an example, plants treated with JA exhibited a decreased egg count in *Pieris rapae* and *P. brassica* in comparison to those feeding on plants that were not treated^118^.

In our investigation, we noted substantial decreases in both larval weight and the survival rate of fall armyworms reared on maize leaves treated first with jasmonic acid followed by salicylic acid, in contrast to the control group. This corroborates earlier findings^80^, where *Helicoverpa armigera* exhibited diminished larval mass when consuming MeJA and SA-treated groundnut leaves. Similar outcomes were evident in the other work^83^ regarding sorghum defenses against stem borers. Notably, an experiment focused on the response to *Spodoptera litura* infestation and jasmonic acid application across three groundnut genotypes^85^ (unveiled significant modulations in physiological attributes, insect weight accrual, and larval survival^85^ reported that externally applied JA can instigate insect resistance mechanisms and foster growth by mitigating plant damage in groundnut plants."

FH-1046 exhibited markedly reduced plant damage compared to YH-1898 amid an infestation of armyworms. Visible alterations in leaf size, shape, and color, including distinct 'window-feeding' damage, underscored the plants' stress due to larvae feeding behavior. These morphological shifts serve as pivotal early indicators of infestation. However, an intriguing contrast emerged in the context of elicitor treatments, revealing a conspicuous decline in larval weight and the production of web structures in both genotypes, especially when compared to untreated plants.

Changes of defense gene expression in SA/JA treated or FAW infected leaves of both cultivars in the present study are associated with the defense related genes expressed by the activation of SA and JA/SA defense pathways respectively as denoted in (Figs. 8, 9). Activation of JA-dependent genes by FAW could be an important part of a plant defense response. The difference in expression level in the maize cultivars may be due to variation in phenotype and metabolic secondary metabolite production level after application of different phytohormones and FAW infestation**.** It has been reported that the roles of SA, JA, mediated signaling pathways vary in different plant-pathogen interactions^40–42^. Pathogenesis-related (*PR)* genes encode some of the plant proteins that break down cellular constituents of pathogens and help in signaling. According to some researchers, PR-1 and an acidic, apoplastic form of b-1,3-glucanase (*BGL2*) are excellent *PR* gene markers of SA-dependent induction in Arabidopsis and expression is interrelated to systemic acquired resistance to further pathogen infection. According to the finding, insect infection has been shown to induce localized and plant-wide increases in mRNA transcription and enzyme activity of numerous pathogenesis related proteins. PR5 group contains acidic, neutral and very basic members with extracellular and vacuolar localization^120^. SA/JA-inducible Phe-ammonia lyase (PAL) also increased in leaves of maize infested with FAW and treated with phytohormones during our study. PAL is a vital enzyme in the formation of phenolic contents which facilitate the activation of carbohydrates at the site of infection. PAL enzyme lead to the biosynthesis of salicylic acid^121^. Overall, in the present study JA applied to maize plants ameliorated the adverse effects of biotic stress on the plants by counteracting oxidative damage, activation of biosynthetic pathway, up regulation of defense genes hence improving the resistance and increasing growth and development as compared to the non-treated plants.

FH-1046 displayed a higher mortality rate among the insects compared to YH-1898, coupled with a reduction in larval weight for those feeding on FH-1046 versus YH-1898. The variance in larval weights across treatments, encompassing JA + FAW, SA + FAW, and FAW, was starkly apparent. This reduction in larval weights, coupled with increased plant resistance, diminished survival rates, and minimized plant damage, is hypothesized to stem from an upsurge in defensive compounds and secondary metabolites within the resistant genotypes, potentially augmented by the application of phytohormones^48,122^.

## Conclusion

Fall Armyworm (FAW) infestation on maize treated with phytohormones revealed compelling insights. The application of JA exhibited a remarkable upsurge in morphological parameters, photosynthetic pigments, and the accumulation of osmolytes, such as proline and potent defense compounds such as phenolics, highlighting its potential in enhancing plant resilience against FAW infestation as indicated by the enhanced expression level of JA mediated genes. Notably, the heightened antioxidant activity observed in JA-treated plants signifies a bolstered defense mechanism against oxidative stress induced by FAW. On the other hand, a contrasting trend emerged with the reduction in detoxification enzymes, indicative of a nuanced trade-off between defense strategies. Conversely, SA treatment, though impactful, demonstrated comparatively lower efficacy in elevating the studied parameters, suggesting a differential mode of action in mitigating FAW stress. The findings unequivocally establish a pivotal role for JA in fortifying maize plants against FAW, evident through reduced plant damage and larval weight. Importantly, FAW infestation induced detrimental effects, diminishing various parameters while intensifying oxidative stress markers and detoxification enzyme levels. This comprehensive analysis underscores the potential of JA application as a strategic approach to bolster plant defense mechanisms, offering a promising avenue for agricultural strategies aimed at mitigating the adverse effects of FAW infestation on maize crops. Moreover, the significant modulation observed in growth, pigment levels, osmolytes accumulation, and antioxidant activity with JA application underscores its pivotal role in restoring redox homeostasis, thereby fortifying the maize plants against the oxidative onslaught triggered by Fall Armyworm infestation.

## Data Availability

The data collected and analyzed for this article has been included in manuscript. However, if some other details are required, can be had from corresponding authors.
